# Analysis of enriched rare variants in *JPH2*-encoded junctophilin-2 among Greater Middle Eastern individuals reveals a novel homozygous variant associated with neonatal dilated cardiomyopathy

**DOI:** 10.1038/s41598-019-44987-6

**Published:** 2019-06-21

**Authors:** Edward G. Jones, Neda Mazaheri, Reza Maroofian, Mina Zamani, Tahereh Seifi, Alireza Sedaghat, Gholamreza Shariati, Yalda Jamshidi, Hugh D. Allen, Xander H. T. Wehrens, Hamid Galehdari, Andrew P. Landstrom

**Affiliations:** 1 Department of Pediatrics, Section of Pediatric Cardiology, Baylor College of Medicine, Houston, Texas United States; 2 Department of Genetics, Faculty of Science, Shahid Chamran University of Ahvaz, Ahvaz, Iran; 3 Narges Medical Genetics and Prenatal Diagnosis Laboratory, Kianpars, Ahvaz, Iran; 4 Molecular and Clinical Sciences Institute, St George’s University of London, London, United Kingdom; 5 Cardiovascular Research Institute, Baylor College of Medicine, Houston, Texas United States; 6 Diabetes Research Center, Health Research Institute, Ahvaz Jundishapur University of Medical Sciences, Ahvaz, Iran; 7 Department of Molecular Physiology and Biophysics, Department of Medicine, Section of Cardiology, Center for Space Medicine, Baylor College of Medicine, Houston, Texas United States; 8 Present Address: Department of Pediatrics, Division of Cardiology, Duke University School of Medicine, Durham, North Carolina United States

**Keywords:** Disease genetics, Cardiovascular genetics

## Abstract

Junctophilin-2 (JPH2) is a part of the junctional membrane complex that facilitates calcium-handling in the cardiomyocyte. Previously, missense variants in *JPH2* have been linked to hypertrophic cardiomyopathy; however, pathogenic “loss of function” (LOF) variants have not been described. Family-based genetic analysis of GME individuals with cardiomyopathic disease identified an Iranian patient with dilated cardiomyopathy (DCM) as a carrier of a novel, homozygous single nucleotide insertion in *JPH2* resulting in a stop codon (JPH2-p.E641*). A second Iranian family with consanguineous parents hosting an identical heterozygous variant had 2 children die in childhood from cardiac failure. To characterize ethnicity-dependent genetic variability in *JPH2* and to identify homozygous *JPH2* variants associated with cardiac disease, we identified variants in *JPH2* in a worldwide control cohort (gnomAD) and 2 similar cohorts from the Greater Middle East (GME Variome, Iranome). These were compared against ethnicity-matched clinical whole exome sequencing (WES) referral tests and a case cohort of individuals with hypertrophic cardiomyopathy (HCM) based on comprehensive review of the literature. Worldwide, 1.45% of healthy individuals hosted a rare *JPH2* variant with a significantly higher proportion among GME individuals (4.45%); LOF variants were rare overall (0.04%) yet were most prevalent in GME (0.21%). The increased prevalence of LOF variants in GME individuals was corroborated among region-specific, clinical WES cohorts. In conclusion, we report ethnic-specific differences in *JPH2* rare variants, with GME individuals being at higher risk of hosting homozygous LOF variants. This conclusion is supported by the identification of a novel JPH2 LOF variant confirmed by segregation analysis resulting in autosomal recessive pediatric DCM due to presumptive JPH2 truncation.

## Introduction

Dilated cardiomyopathy (DCM) is defined as a primary myocardial disorder characterized by ventricular dilation and impaired contractility not explained by abnormal loading conditions or ischemic insult^1^. DCM is attributable to both genetic and nongenetic causes and is found to be familial in about 25 to 50% of patients^2–4^. Of these familial cases, up to 37% have a clinically relevant genetic variant, leaving the majority of cases genetically unexplained^5^. DCM is a genetically heterogenous disease with disease-associated genes ranging from sarcomeric structure to metabolic etiologies to MAPK pathways. One such category includes genes encoding for proteins in calcium (Ca^2+^)-signaling, and -sensitive, pathways, such as *JPH2*-encoded junctophilin type 2 (JPH2)^6^.

JPH2 is a striated muscle-specific protein and a critical member of the junctional membrane complex (JMC) that regulates myocardial excitation-contraction (E-C) coupling^7,8^. Cardiac contraction is dependent on efficient E-C coupling which is mediated by Ca^2+^. Ca^2+^ influx through voltage-gated L-type calcium channels in the t-tubular plasma membrane triggers further Ca^2+^ release via the ryanodine receptor 2 in the sarcoplasmic reticulum^9,10^. JPH2 anchors the junction between t-tubular sarcolemma and the sarcoplasmic reticulum as well as stabilizes the ryanodine receptor 2^8,11,12^. Early studies have suggested a role for JPH2 in the development of DCM, with loss of normal JPH2 expression during pathologic remodeling^13–16^. The importance of JPH2 in the structural and functional integrity of the JMC is further reinforced clinically by the observation of disease-associated variants in *JPH2* found in a small number of patients with cardiomyopathy as well as atrial fibrillation^11,17–20^.

The clinical expansion of broad genetic sequencing, such as whole exome sequencing (WES), has allowed for rare causes of cardiomyopathy to be identified and research-based exome sequencing has identified rare variants in disease-associated genes. Despite a greater incidence of autosomal recessive disease-causing variants, regions such as the Greater Middle East (GME) remain underrepresented and understudied^21–23^. In this study, we identify two Iranian families hosting a novel loss-of-function (LOF) *JPH2* variant that, when homozygous, was associated with DCM and death in early childhood from cardiac failure. We then systematically examine the background frequency of rare variants of *JPH2* in regionally diverse populations as well as clinical WES sequencing referrals, including those from the GME. We conclude that there are ethnic-specific differences in rare *JPH2* variants, with GME individuals being at higher risk of hosting homozygous LOF variants.

## Methods

### Study cohorts

This research study was approved by the Baylor College of Medicine and the Ahvaz Jundishapur University of Medical Sciences Institutional Review Boards. For genetic studies involving cohorts, informed consent was waived. For WES studies, informed consent was obtained. All experiments were performed in accordance with relevant guidelines and regulations.

#### Population-based control cohorts

Worldwide, population-based, control variants were derived from the Genome Aggregate Database (gnomAD) made up of 138,632 individuals as well as regionally-specific cohorts^24^. While the gnomAD database is comprised partly of various disease-specific cohorts in addition to population genetics studies, it excludes individuals known to have severe pediatric disease as well as severe disease in their first-degree relatives; therefore, we utilized these individuals as “control” or “reference” alleles. Furthermore, although this database includes many geographically distinct cohorts, the GME is under-represented within gnomAD and individuals from this region are not clearly delineated. Thus, variants of ostensibly healthy individuals from GME were derived from the GME Variome (*N* = 1,111)^21^ and Iranome (*N* = 800)^25^, respectively. The GME Variome includes individuals from a large collection of Arab and non-Arab Muslim countries (Morocco, Algeria, Tunisia, Libya, Egypt, Turkey, Syria, Lebanon, Israel, Saudi Arabia, Iraq, Qatar, Kuwait, Yemen, UAE, Iran, Oman, and Pakistan), and excludes individuals with a genetic kinship coefficient suggestive of a high degree of relatedness. Given overlap in the population of GME Variome and the African/African American population of gnomAD, Iranome was included to control for the population solely from Iran. For population frequency calculations of “rare variants,” a minor allele frequency (MAF) of <0.01 was utilized. In total, 140,543 individuals across these 3 cohorts were included as controls.

#### Baylor and Iranian whole exome sequencing cohorts

Given widespread advancement in clinical WES testing, we determined the frequency of *JPH2* variants in 2 clinical WES referral cohorts. The Baylor clinical WES cohort has been previously described^26^ and is derived from WES variant data compiled by Baylor Genetics Laboratories^27^. This cohort was comprised of 7,066 probands referred for WES genetic testing to the Baylor Genetics Laboratories (Houston, Texas, United States) independent of referral diagnosis or indication for genetic testing. Individuals included in this cohort were genetic testing probands. Demographic and clinical referral information was abstracted. Genetic information from samples derived for platform validation studies, or from oncological samples, was excluded. For GME-matched WES referral variants, a cohort of 823 clinical WES referrals from Iran were analyzed. Variants included in these WES cohorts were (1) identified in the coding nucleotide sequence or predicted splice junction of an HCM-associated gene locus, (2) potential splice donor or splice acceptor-effecting variants located within the first four nucleotides near the splice junction, and (3) deemed “pathogenic”, “likely pathogenic”, or “variant of uncertain significance” (VUS) at the time of genetic testing according to the American College of Medical Genetics (ACMG) criteria/interpretation guidelines^28,29^, and (4) included on the clinical report sent, or made available, to the referring provider. Variants excluded from this study were: (1) interpreted as “not pathogenic” at the time of genetic testing, (2) non-splice site intronic variants, (3) 5′or 3′ untranslated region variants, or (4) synonymous variants. To account for changes in evidence of pathogenicity since the initial variant identification and classification at the time of genetic testing, each variant in the Iranian and Baylor WES cohorts was subjected to further verification for pathogenicity via ClinVar aggregate records^30^. All variants classified as “benign” or “likely benign” as of January 23, 2019 were excluded.

#### Pathogenic variant cohort

To determine the prevalence of cardiomyopathy-associated *JPH2* variants, previously published studies were utilized^11,17,18^. A combined prevalence from these cohort-based studies was created. Inclusion criteria were (1) associated with a proband/family with a clinical diagnosis of hypertrophic or dilated cardiomyopathy, (2) absence of a compound variant deemed to be a likely disease-associated variant, (3) absence of the variant in reference/control alleles, and (4) identified in a cohort-based study. Variants obtained from non-cohort-based studies that otherwise met inclusion criteria were utilized for non-prevalence analyses.

### Whole exome sequencing

Clinical WES testing was conducted as previously described for the Baylor WES cohort^27^. Briefly, extracted DNA was subjected to an in-house exome capture platform (VCRome version 2.1) and sequenced using either an Illumina Genome Analyzer IIx platform or the Illumina HiSeq 2000 platform. Samples were additionally analyzed by an Illumina HumanExome-12 v1 cSNP array for quality-control assessment of exome data, as well as for detecting large copy-number variants and regions of absence of heterozygosity. Iranian clinical WES testing utilized commercial platforms (Beijing Genomic Institute, Shenzhen, China; Macrogen, Seoul, South Korea)^30^. WES performed on DNA from the proband of the first family; the second family (Family 2) had WES performed on parents due to unavailability of samples from deceased children. Haplotype analysis was performed as previously described^31^. Presence or absence of putatively pathogenic variants were compared against the 1000 Genomes Project^32^, NHLBI GO Exome Sequencing Project, gnomAD, as well as the ethnically-matched GME Variome and Iranome. Confirmation of the WES-identified JPH2-p.E641* variant was conducted in the probands of each kindred using direct Sanger sequencing. Further, variant positivity was evaluated in all kindred using Sanger sequencing.

### Nomenclature

LOF variants were defined as variants that are predicted to cause a protein loss-of-function including nonsense (early termination), insertion/deletion (both in-frame and out-of-frame), and predicted canonical splice site variants. Among WES variants, designations of pathogenic, likely pathogenic, or VUS were based on the designation at the time of WES testing. Variant annotations were based on established nomenclature^33^.

### Sequence homology and domain mapping

JPH2 consensus primary sequence (NM_020433, NP_065166) was utilized from the Ensembl browser^34^. Variants were mapped along the protein topology. Primary sequence conservation among 56 independent JPH2 orthologues was compared to determine degree of conservation across species using the National Center for Biotechnology Information (NCBI).

### Statistics

Statistical results were expressed as mean with variance expressed as standard deviation or median and interquartile range (brackets), as appropriate. Variance of prevalence/proportion was expressed as the exact 95% confidence interval around proportion when statistical comparisons were made (brackets). Comparisons were made by Student’s t-Test, Fisher’s Exact test, Chi-Square with Yates Correction, as appropriate using OpenEpi^35^. Statistical significance threshold was set at *P* < 0.05.

## Results

### Identification of a homozygous JPH2-p.E641* variant in an infant with DCM

**Figure 1:**
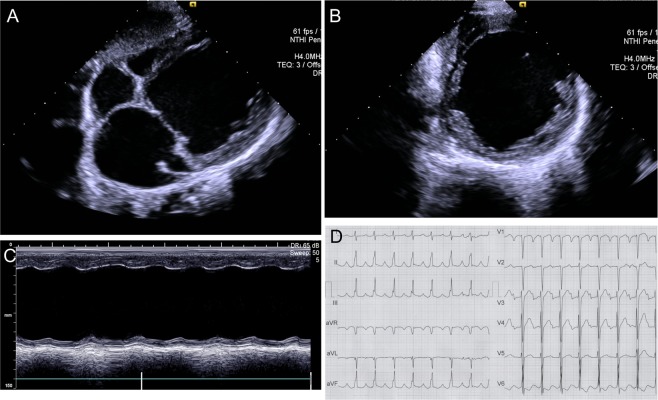
(**A**–**C**) Transthoracic echocardiography images of the JPH2-p,E641* homozygous variant-positive proband demonstrating a severely dilated left ventricle with reduced systolic function and D, 12-lead ECG demonstrating bi-atrial dilation, PR-prolongation, interventricular conduction delay, and T wave abnormalities.

**Figure 2:**
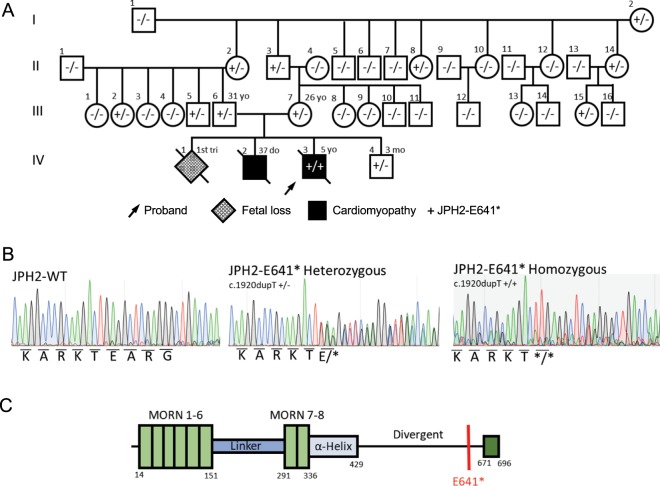
(**A**) Family 1 pedigree of the JPH2-p.E641* homozygous variant-positive proband and extended kindred. Arrow denotes proband; gray fill, fetal loss; black fill, dilated cardiomyopathy; diagonal line, deceased. (**B**) Sanger sequence chromatograms depicted wild type JPH2 as well as JPH2-p.E641* heterozygous and homozygous sequencing. (**C**) Linear topology with protein functional domains of JPH2 are depicted with location of p.E641* variant (red line) in the divergent region.

The proband demonstrated severe left ventricular (LV) dilation with rapidly declining systolic function as an infant. He initially presented at 20 months of age after being hospitalized with fever, tachypnea, and restlessness. Imaging tests, including chest radiographs, electrocardiogram, and echocardiography, confirmed the diagnosis of DCM. By 4.5 years of age, LV systolic function was severely depressed with medically-refractory heart failure (Fig. 1A–C). A representative electrocardiogram demonstrating PR-prolongation, conduction delay, and T-wave abnormalities is depicted in Fig. 1D. The patient was referred for cardiac transplantation; however, the patient ultimately died at 5 years of age while awaiting transplant. This child was the offspring of consanguineous parents (first cousins) who had 2 affected sons. The pedigree is depicted in Fig. 2A. The mother (III-7) had 4 total pregnancies, with 1 spontaneous abortion at 8 weeks (IV-1), and 3 live births. The second affected pregnancy was an infant boy (IV-2) who presented with sudden fever, poor feeding, jaundice, and hypotonia as a neonate. He had cardiomegaly, clinical features consistent with cardiomyopathy, and died at 37 days of age after a cardiorespiratory arrest. Subsequently, the mother gave birth to the proband (IV-3).

Genetic testing for genes canonically associated with DCM were negative for likely pathogenic variants. WES was performed and the proband was found to host a homozygous single nucleotide insertion (c.1920dupT) resulting in a premature stop codon (JPH2-p.E641*) (Fig. 2B,C; Supp. Fig. 1). The proband hosted no other pathogenic variants in currently known monogenic disease-causing genes identified on WES. This *JPH2* variant is novel and absent in 299,100 reference alleles derived from healthy individuals. JPH2-p.E641* localizes to the C-terminal divergent region in an area of low sequence homology and relatively high variation. Segregation analysis was performed for the variant to determine the mode of inheritance. Although no genetic information was available for the family’s first and second children (IV-1, IV-2), both parents and their fourth child (III-6, III-7, IV-4) were found to be heterozygous for JPH2-p.E641*. Additionally, this variant was found in the heterozygous state in 8 of 32 healthy family members of the proband who were available for genetic screening. These findings suggest an autosomal recessive mode of inheritance with functional genetic truncation of *JPH2* associated with neonatal cardiomyopathy.

### JPH2-p.E641* variant possibly associated with rapid ventricular failure in neonatal Ebstein anomaly

**Figure 3:**
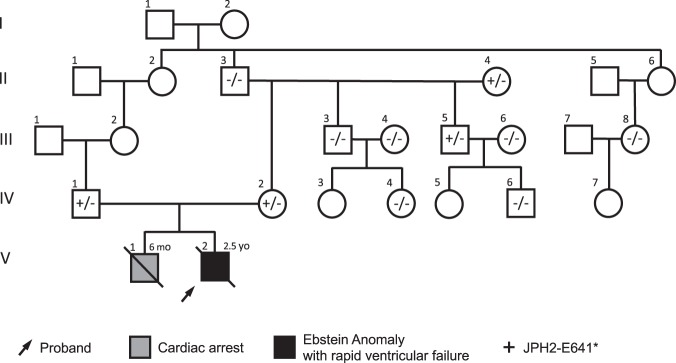
Family 2 pedigree of the GME family with both consanguineous parents hosting heterozygous JPH2-p.E641* variants. Arrow denotes proband; gray fill, cardiac arrest; black fill, Ebstein anomaly with rapidly progressive dilated cardiomyopathy; diagonal line, deceased.

Supporting the hypothesis that homozygous JPH2-p.E641* is associated with cardiac failure, we identified a second consanguineous Iranian family with this variant (Fig. 3). This family was seemingly unrelated to the previous family although they shared a similar ethnic background (Lor – a group of Iranian people found predominantly in southwest Iran). The first child of the mother and father (first cousins once-removed) presented at 6 months of age with fever, restlessness, and poor feeding following a sandstorm. His clinical status continued to decline after admission, and he eventually died from in-hospital cardiac arrest 3 days later. The second pregnancy was prenatally diagnosed with Ebstein anomaly and the child initially did well until 2 years of age when he underwent tricuspid valve annuloplasty and atrial septal defect closure. Post-operatively, he had episodes of respiratory distress and gradually developed fluid-filled blisters on his skin and had generalized edema involving hands, feet, face and abdomen. He developed progressive ventricular failure and died at 2.5 years of age due to a cardiac arrest. DNA was not obtained on either child; however, both parents were found to be heterozygous for JPH2-p.E641*. Extended segregation analysis identified 2 heterozygous carriers out of 9 additional healthy family members of the proband who did not have cardiomyopathy or history of cardiac disease and were able to undergo genetic testing. Haplotype analysis using rare variants identified in the WES data from the proband of Family 1, and the parents of Family 2 showed an identical haplotype containing the *JPH2* variant, indicative of a founder effect.

### *JPH2* demonstrates ethnic-specific variability in genetic sequence

Given our findings of a truncating LOF *JPH2* variant associated with cardiac death early in childhood in two GME families, we next sought to characterize the differences in genetic variation of *JPH2* amongst ethnically distinct populations. We first examined the frequency of rare *JPH2* variants in several population-based genomic studies of control individuals. Among 138,632 individuals genotyped in the gnomAD cohort, there were 2,015 variants with a MAF < 0.01 that contributed to a total rare variant frequency of 1.45% [1.39–1.52]. When divided by regional subtypes, the highest prevalence of rare *JPH2* variants was found in African American/African individuals (5.03% [4.65–5.43]) followed by European (Finnish) (1.74% [1.51–1.96]). Given the underrepresentation of GME individuals within gnomAD, and recent evidence of high genetic variation and autosomal recessive variants^21^, we next evaluated 2 additional GME-specific databases. Of the 1,111 individuals in the GME Variome cohort, there were 50 variants with a MAF < 0.01 leading to a total variant frequency of 4.50% [3.28–5.72]. Due to potential overlap between the GME Variome population and African/African American subgroup of gnomAD, the Iranome cohort was also included. The Iranome cohort demonstrated 35 variants found within 800 individuals providing a prevalence of 4.38% [2.96–5.79]. The GME Variome and Iranome cohorts each demonstrated a higher overall prevalence of *JPH2* variants than any gnomAD subgroup other than African American/African (*P* < 0.001). There was no difference between GME Variome and Iranome *JPH2* variant frequency.

**Figure 4:**
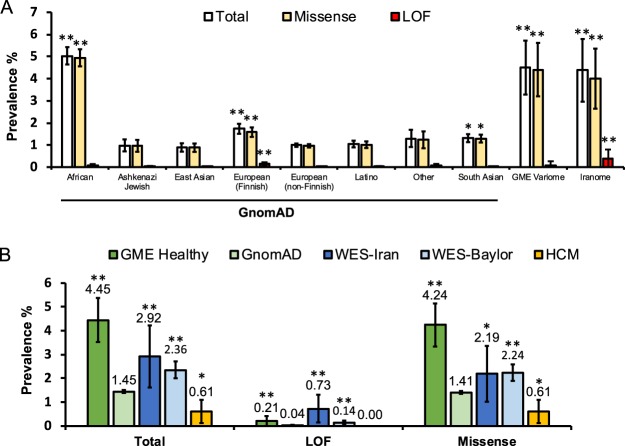
(**A**) Bar graph comparing the frequencies of rare *JPH2* variants in ostensibly healthy individuals. The gnomAD cohort is broken down into 8 ethnic subgroups. GME Variome and Iranome represent 2 independent Middle Eastern cohorts. No fill, total frequency; tan, missense variant frequency; red, LOF variant frequency. Error bars denote 95% CI; *P < 0.05, **P < 0.001 compared to European (Non-Finnish). (**B**) Bar graph comparing the frequencies of *JPH2* variants in a GME Healthy Cohort (GME Variome + Iranome), an ostensibly healthy population (gnomAD), a cohort of clinical WES referrals at Baylor College of Medicine (WES), a cohort of clinical WES referrals from Iran (WES-Iran), and a population of those diagnosed with HCM. Error bars denote 95% CI; *P < 0.05, **P < 0.001 compared to gnomAD.

Given the high prevalence of rare variants within control individuals, we next evaluated these cohorts for rare LOF variants. The incidence of LOF variants was rare within the gnomAD cohort with a prevalence of 0.04% [0.03–0.05]. In comparison, the Iranome cohort contained 3 identified LOF variants for an overall prevalence of 0.375% [0.00–0.80], nearly 10-fold the prevalence seen in gnomAD cohort. This was significantly higher than all regional groups except European (Finnish) (0.16%, [0.09–0.22]) (*P* < 0.001). The GME Variome hosted 1 LOF variant (0.09% [0.00–0.27]). For the purposes of comparison, both the GME Variome and Iranome were combined into a “GME Healthy Cohort” that had an overall *JPH2* variant prevalence of 4.45% [3.52–5.37], with a LOF variant prevalence of 0.21% [0.01–0.41]. All LOF variants in control cohorts were heterozygous. There was a single truncating LOF variant noted in the GME Healthy Cohort. However, there were 25 total individuals in the gnomAD cohort (0.02% [0.01–0.03]) hosting heterozygous truncating variants, 18 of which were Finnish. There were no homozygous truncating variants noted in any control cohort. These results are summarized in Fig. 4A. Taken together, these results indicate a greater burden of *JPH2* variation in healthy individuals from GME regions, particularly in LOF variants.

### Variants in *JPH2* demonstrate ethnic-specific variability among clinical WES referrals

Due to widespread advancement in clinical WES testing, we next compared these findings to the frequency of identified variants within regionally-matched cohorts of clinical WES referrals. Among 7,066 unrelated probands undergoing clinical WES testing at Baylor College of Medicine, 167 probands hosted a *JPH2* variant (2.36% [2.01–2.72]), including 10 probands with LOF variants (0.14% [0.05–0.23]). These were both modestly higher than the total variant prevalence and LOF variant prevalence observed in the gnomAD cohort (*P* < 0.001). Similarly, among 823 Iranian clinical WES referrals, 24 probands hosted a rare variant of *JPH2* (2.92% [1.77–4.07]), 6 of which were LOF (0.73% [0.15–1.31]). Among the 6 LOF variants identified within the Iranian WES cohort, two were JPH2-p.E641* variants – one from each of the two families described above. One JPH2-p.E641* variant represents the homozygous affected proband from Family 1, whereas the second variant represents a heterozygous unaffected parent from Family 2 with evidence of two infant deaths related to cardiac failure. All LOF variants identified in clinical WES cohorts are detailed in Table 1. Between these 2 clinical WES cohorts, there was no significant difference in prevalence of total variants; however, the Iranian WES cohort demonstrated a significantly higher prevalence of LOF variants (*P* < 0.001). Overall, this indicates a higher incidence of *JPH2* LOF variants in GME individuals referred for clinical genetic testing in independent region-specific cohorts, corroborating a similar finding in population-based, ostensibly healthy individuals. These results are summarized in Fig. 4B.

**Table 1:** Loss of Function Variants Identified in WES Cohorts.

Nucleotide	Amino Acid	Zygosity
**Baylor WES Cohort**		
c.1778_1779insGGTCCG	p.E593delinsGSE	Het
c.1819_1820insACCGCCCCGCT	p.P607delinsTAPLQAP	Het
c.349_351del	p.117del	Het
c.349_351del	p.117del	Het
c.516_517insAGCAAC	p.G173delinsSNG	Het
c.516_517insAGCAAC	p.G173delinsSNG	Het
c.517_518insAGCAAC	p.G173delinsSNG	Het
c.517_518insAGCAAC	p.G173delinsSNG	Het
c.517_518insAGCAAC	p.G173delinsSNG	Het
c.55del	p.E19fs	Het
**Iranian WES Cohort**		
c.1878_1879insC	p.I627fs	Het
c.1920dupT	p.E641*	Hom^#^
c.1920dupT	p.E641*	Het^##^
c.511_516dupAGCAAC	p.S171_N172dup	Het
c.511_516dupAGCAAC	p.S171_N172dup	Het
c.864_865insACCACC	p.T288_E289insTTT	Het

WES, whole exome sequencing; Het, heterozygous; Hom, homozygous; ^**#**^proband from Family 1; ^**##**^parent from Family 2.

### Frequency of *JPH2* variants among cases of cardiomyopathy

Due to the high background rate of rare variants in *JPH2*, we explored the rate of pathogenic *JPH2* variants found in patients clinically diagnosed with cardiomyopathy. Of the 981 individuals with cardiomyopathy in the HCM cohort, only 6 (0.61% [0.12–1.10]) hosted a *JPH2* variant, all of which were heterozygous missense variants. This was significantly lower than the prevalence demonstrated in gnomAD (1.45%, *P* < 0.05), the GME Healthy cohort (4.45%, *P* < 0.001), and both of the clinical WES cohorts (WES-Iran 2.92%, WES-Baylor 2.36%, *P* < 0.001). No LOF variants were described. These results are summarized in Fig. 4B. Importantly, of the 6 cardiomyopathy-associated variants identified, only 1 was also found to be present in control cohorts (JPH2-A405S, gnomAD MAF = 9.69E-05). None of the 7 cardiomyopathy-associated variants were found in the GME Variome or Iranome cohorts. Taken together, these results suggest that pathogenic JPH2 variants are rare among patients with cardiomyopathy and are largely absent in population and clinical WES-based cohorts.

### Comparison of genetic variability, orthologue identity, and variant topology

**Figure 5:**
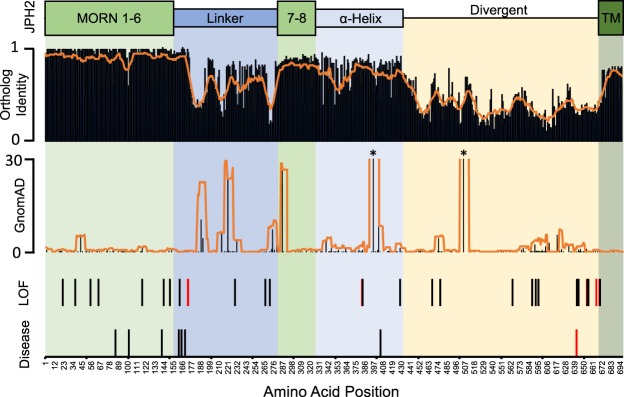
Amino-acid level genetic variability analysis of *JPH2*. Functional domains of *JPH2* are depicted. Ortholog identity map and prevalence of variants in healthy individuals (gnomAD) by amino acid are depicted with orange lines representing rolling averages. Amino acid positions containing radical variants found in ostensibly healthy individuals and disease-causing variants found in those with cardiomyopathies are depicted, with those in red representing variants identified in individuals from the Middle East. MORN, membrane occupation and recognition nexus domain; TM, Transmembrane domain. *MAF exceeding Y-axis.

To characterize the genetic variation found in *JPH2*, amino-acid level sequence homology analysis was performed to correlate with genetic variation. The structure of JPH2 is comprised of 8 membrane occupation and recognition nexus (MORN) domains that associate with the t-tubule at the N-terminus, a C-terminal transmembrane domain that anchors into the sarcoplasmic reticulum, and an alpha-helical domain that spans the junctional space in between^8^. We observed the highest degree of sequence homology in the MORN domains, followed by the transmembrane and alpha-helix domains. There was markedly less sequence homology demonstrated in the linker and divergent domains. We next overlaid the prevalence of all variants in individuals from the gnomAD cohort by amino acid, including those above the MAF threshold, which revealed generally higher levels of genetic variability in regions with less sequence homology, such as the linker region. There were 2 common variants within the gnomAD cohort with MAF > 0.01 included in this analysis, JPH2-A396T (MAF = 0.179) and G505S (MAF = 0.011). Amino acid positions containing LOF variants found in ostensibly healthy individuals as well as disease-associated variants were also mapped. Both the LOF and the disease-associated variants did not appear to localize to areas of high homology nor to areas of high genetic variability. These results are summarized in Fig. 5. Overall, these findings suggest that areas of high sequence variation correspond to areas less conserved across species. However, both cardiomyopathy-associated variants and LOF variants found in ostensibly healthy individuals do not seem to localize to specific “hot spots” on the protein.

## Discussion

In this study, for the first time to our knowledge, we present a novel homozygous LOF *JPH2* variant, JPH2-p.E641*, associated with autosomal recessive DCM in an Iranian family, confirmed by segregation analysis. This finding was further supported by a second Iranian family where both healthy parents carry the same LOF *JPH2* variant and who had 2 children who died in early childhood from cardiac failure, 1 in the context of structural heart disease. In the case of this novel truncating nonsense variant, it is possible that the homozygous loss of the C-terminal transmembrane domain in individuals hosting JPH2-p.E641* causes loss of anchoring to the sarcoplasmic reticulum, resulting in impaired Ca^2+^ handling and increased risk of heart failure early in life. Prior to these observations, only heterozygous missense *JPH2* variants have been linked to a cardiomyopathy phenotype^11,17–19^.

In the setting of these findings, a growing body of evidence has demonstrated that alterations in *JPH2* expression and function can perturb intracellular Ca^2+^ signaling and result in myopathic disease, including HCM and DCM^12,36^. *JPH2*-null mice demonstrate embryonic lethality due to molecular failure of the JMC and ineffective Ca^2+^-signaling needed to sustain EC coupling and cardiac contractility^7,15^. Furthermore, induction of *JPH2* expression silencing in adult mice is associated with rapid deterioration into heart failure with a dilated left ventricle and loss of systolic function^16,37^. When combined with the striking phenotype observed in the 2 families presented here, which mimic these rodent models, our findings support the concept that loss of *JPH2* expression can result in rapid progression of cardiomyopathy and heart failure. Additional studies utilizing *in vitro* and *in vivo* models are needed to fully explore this possible mechanism disease.

The investigation of pathogenic *JPH2* variants in individuals with cardiomyopathies has temporally coincided with a recent increase in clinical utilization of next-generation sequencing modalities. Tools like WES not only increase the sensitivity for detecting genetic variants in atypical clinical presentations, but also have given us a window into the natural variability of human genome and how it varies by geographic and ethnic backgrounds^24,38^. Based on the underrepresentation of GME populations in current large-scale public genome databases, and the relatively high density of genetic disease within the GME population, we sought to examine whether significant genetic variability in *JPH2* existed between GME and other ethnic populations. Remarkably, we found a high burden of genetic variation, including LOF variants, within cohorts representing the GME. The higher prevalence of LOF variants was replicated in clinical genetic testing referrals and may contribute to an overall increased risk of development of *JPH2*-related AR cardiomyopathy in those with GME/Iranian ethnic backgrounds. These observations highlight the critical nature of comparing disease-associated variants with ethnically-matched control alleles. This is reflected in the American College of Genetics and Genomics recommendations to include race-matched control data when interpreting sequence variants^39^.

The variants from WES cohorts in *JPH2* are difficult to interpret, particularly in individuals with a low pre-test probability of disease. Previously there has been evidence to suggest that incidentally identified variants in channelopathies likely represent background genetic variation^26,40^ Both clinical WES cohorts hosted variants at a markedly higher rate than cardiomyopathy cases, which supports the hypothesis that the majority of these *JPH2* variants also represent background noise. The requirement of the truncating variant JPH2-p.E641* to be homozygous in order to produce clinical disease also suggests that previously described heterozygous missense variants may result in disease through a dominant-negative effect^41^. However, the challenge in clinical evaluation of children who may develop a cardiomyopathy phenotype later in life must be recognized. Furthermore, the presence of variants in phenotype-negative individuals does not necessarily exclude variant pathogenicity, as is seen in *TTN* truncating variants that have been shown to cause DCM despite their presence in up to 1% of the general population^42^. This highlights the need for more research to improve identification of susceptibility alleles and the role that missense *JPH2* variants play in contributing to cardiomyopathies.

Previous studies in arrhythmogenic disorders have demonstrated the value of mapping variant locations in assessing for risk of pathogenicity, suggesting that some regions of disease-causing genes may have elevated signal-to-noise ratios^26,43^. In an effort to characterize the genotype-phenotype mechanism of this study’s novel LOF variant, we stratified *JPH2* variant analysis at the amino acid level and compared it to other previously identified variants. We found that both LOF and disease-causing variants did not cluster in areas of either increased homology or increased variance, nor did they localize to a specific protein motif. Combined with the many healthy Iranian family members that hosted a single copy of JPH2-p.E641*, this reinforces the likelihood that a heterozygous LOF variant would be insufficient to cause disease. Additionally, while the number of pathogenic and LOF variants are relatively small, these results suggest that there are no specific disease-associated “hotspot” domains in *JPH2*.

This study has several limitations. First, the supporting evidence that the second Iranian family provides in the potential pathogenicity of JPH2-p.E641* is limited by the lack of genotype on any of the diseased offspring noted. However, the presence of additional healthy heterozygous individuals, when combined with the incidence of multiple presumed cardiac deaths in childhood in the offspring of two heterozygous parents, warranted inclusion of the family in the study. Second, this study uses a MAF of <0.01 as an upper threshold for inclusion of rare variants in control cohorts. Though a MAF threshold of <0.0001 has previously been validated for rare cardiomyopathy-associated variants^44^, this upper bound was not feasible given the smaller size of the GME Variome and Iranome cohorts. The large difference in the size of these cohorts makes comparison challenging and is a result of the underrepresentation of GME individuals in larger genomic databases. This emphasizes the need for further large-scale investigation into this population.

## Conclusions

Our findings add to the growing evidence that variants in *JPH2* play a role in cardiomyopathy; and suggest that this novel biallelic truncating variant can give rise to severe, early-onset cardiomyopathy. Given the higher prevalence of LOF variants identified in ethnically-matched controls, as well as evidence to suggest that heterozygous LOF variants are insufficient to cause disease, it is likely that this proband’s risk of disease was exacerbated by their ethnic background.

## Supplementary information

SupFig1

## Data Availability

JPH2-p.E641* proband information has been uploaded to PhenomeCentral^45^ (https://www.phenomecentral.org) under ID number P0008365.
