# Supplementary material for: Analysis of enriched rare variants in JPH2-encoded junctophilin-2 among Greater Middle Eastern individuals reveals a novel homozygous variant associated with neonatal dilated cardiomyopathy
Source: Sci Rep. 2019 Jun 21;9:9038. doi: 10.1038/s41598-019-44987-6 (PMC6588559; doi:10.1038/s41598-019-44987-6)
Supplement: Supplementary file 1 — SupFig1 [file 41598_2019_44987_MOESM1_ESM.docx]

**Analysis of enriched rare variants in *JPH2*-encoded junctophilin-2 among Greater Middle Eastern individuals reveals a novel homozygous variant associated with neonatal dilated cardiomyopathy**

Edward G. Jones MD^1,^**^#^**, Neda Mazaheri MSc^2,3,^**^#^**, Reza Maroofian PhD^4^, Mina Zamani MSc^2,3^,

Tahereh Seifi MSc^2,3^, Alireza Sedaghat MD^6^, Gholamreza Shariati MD, PhD^3^, Yalda Jamshidi^4^, Hugh D. Allen MD^1,5^, Xander H. T. Wehrens MD, PhD^5,7^, Hamid Galehdari PhD^2,^*,

Andrew P. Landstrom MD, PhD ^1,5,8,^*

^1^ Department of Pediatrics, Section of Pediatric Cardiology, Baylor College of Medicine, Houston, Texas, United States

^2^ Department of Genetics, Faculty of Science, Shahid Chamran University of Ahvaz, Ahvaz, Iran

^3^ Narges Medical Genetics and Prenatal Diagnosis Laboratory, Kianpars, Ahvaz, Iran

^4^ Molecular and Clinical Sciences Institute, St George’s University of London, London, United Kingdom

^5^ Cardiovascular Research Institute, Baylor College of Medicine, Houston, Texas, United States

^6^ Diabetes Research Center, Health Research Institute, Ahvaz Jundishapur University of Medical Sciences, Ahvaz, Iran

^7^ Department of Molecular Physiology and Biophysics; Department of Medicine, Section of Cardiology; Center for Space Medicine, Baylor College of Medicine, Houston, Texas, United States

^8^ Department of Pediatrics, Division of Cardiology, Duke University School of Medicine, Durham, North Carolina, United States (current institution)

**^#^** Authors contributed equally and are co-equal first authors

***** Authors contributed equally and are co-corresponding authors.

**Correspondence:**

Hamid Galehdari, PhD

Andrew P. Landstrom, MD, PhD

**SUPPLEMENTAL MATERIALS**

**SUPPLEMENTAL FIGURE**

JPH2-E641* Homozygous

c.1920dupT

**
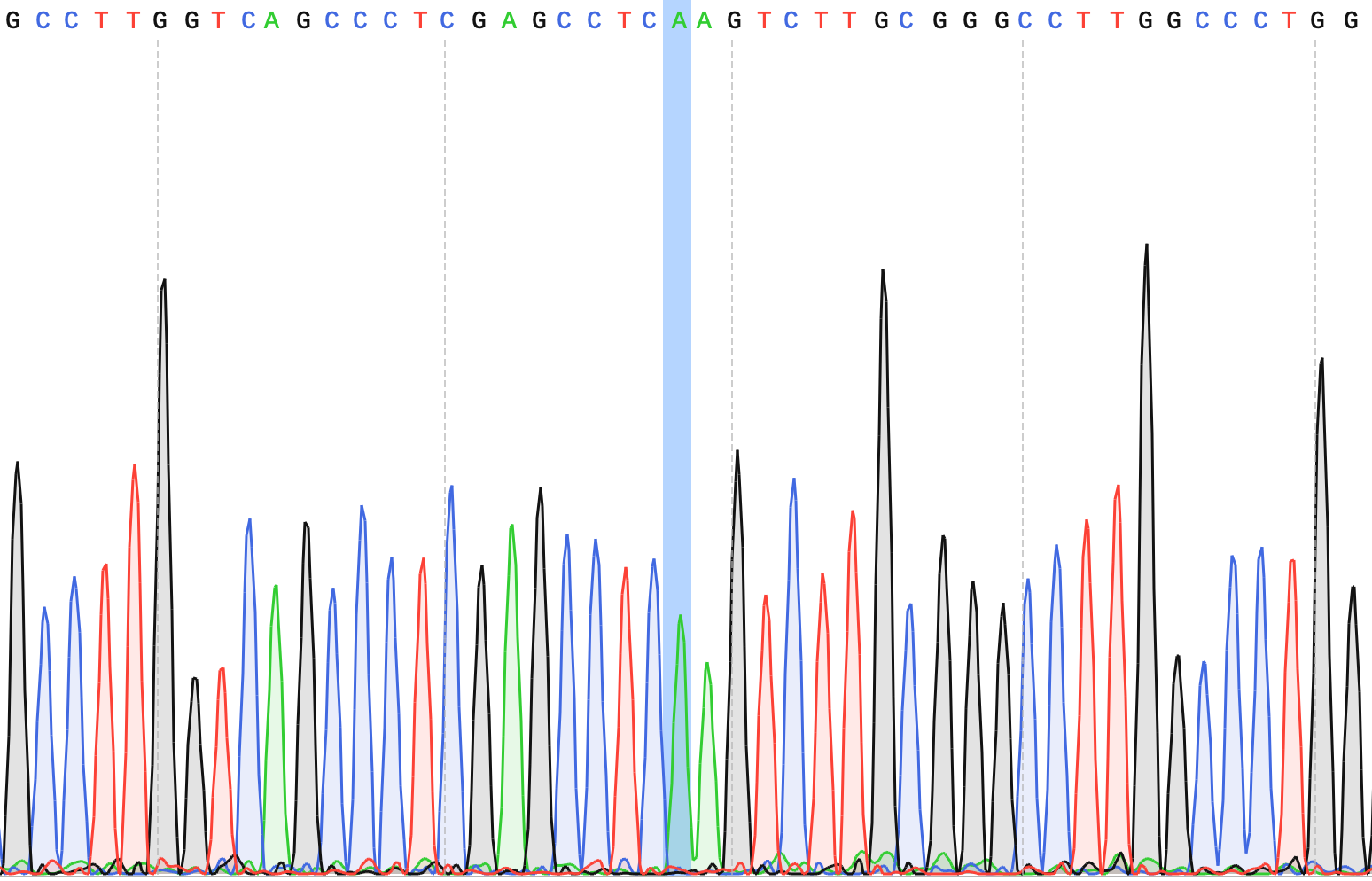
**

**SUPPLEMENTAL FIGURE 1:** Sanger sequencing chromatogram of Family 1 proband, hosting the JPH2-p.E641* homozygous variant, using a reverse sequencing primer. Blue highlighting denotes the homozygous T duplication creating the identified mutation.
